# Pattern of neurological diseases in adult outpatient neurology clinics in tertiary care hospital

**DOI:** 10.1186/s13104-017-2873-5

**Published:** 2017-11-02

**Authors:** Safia Awan, Saad Shafqat, Ayeesha Kamran Kamal, Aziz Sonawalla, Sarwar Siddiqui, Fowzia Siddiqui, Mohammad Wasay

**Affiliations:** 1 Section of Neurology, Department of Medicine, Aga Khan University Hospital Karachi, Karachi, Pakistan; 2 Department of Neurology, Aga Khan University, Stadium Road, Karachi, 74800 Pakistan

**Keywords:** Neurological disorders, Developing countries, Epidemiology, Clinical pattern

## Abstract

**Objectives:**

The burden of neurological diseases in developing countries is rising although little is known about the epidemiology and clinical pattern of neurological disorders. The objective of this study was to understand the burden of disease faced by neurologists a in tertiary care setting.

**Results:**

A prospective observational study was conducted of all presentations to neurology clinics at Aga Khan University Hospital Karachi over a period of 2 years. A total of 16,371 out-patients with neurological diseases were seen during the study period. The mean age of the study participants were 46.2 ± 18.3 years and 8508 (52%) were male. Headache disorders were present in 3058 (18.6%) of patients followed by vascular diseases 2842 (17.4%), nerve and root lesions 2311 (14.1%) and epilepsies 2055 (12.5%). Parkinson’s disease was more prevalent in male participants 564 (70.8%) as compared to female 257 (62.1%) (*p* = 0.002). Migraines and vertigo disease were more diagnosed in females as compared to males. Epilepsies were seen more in younger age groups. Parkinson’s disease was seen in 50.9% of participants between the ages of 45 and 65 years, and the frequency increased with age.

**Electronic supplementary material:**

The online version of this article (10.1186/s13104-017-2873-5) contains supplementary material, which is available to authorized users.

## Introduction

Neurological disorders (NDs) are increasing because of demographic and epidemiologic changes occurring in both developed and developing countries. Neurological disorders accounted for 3.0% of global disability-adjusted life-years (DALYs); mainly due to epilepsy and migraines. While in some regions dementia was a major cause, at the global level NDs accounted for 11.3 million DALYs [1]. The overall burden of neurological disorders significantly increases among adults in low- and middle-income countries (LMICs) [2].

Regional differences in neurologic disorders and stroke related risk of death are smaller at older ages than at younger ages, ranging from around 40 to 60% in the developed countries and 70% in sub-Saharan Africa [1, 3, 4]. The Global Burden of Disease 2010 data suggests that non communicable diseases (NCDs) and injuries account for 77% of age standardized deaths in Pakistan [5].

The burden of neurological diseases in developing countries is rising and it is important to know the disease prevalence and pattern based on geographical, social, cultural, religious, and ethnic factors. A population based study conducted in India found 3355 individuals with neurological disorders per 100,000 populations. The most frequent disorders were headache, febrile convulsions, epilepsy, stroke and mental retardation [6]. A study conducted in Saudi Arabia showed that the crude prevalence of neurological disorders was 131/1000 population. Headache, epilepsy, febrile convulsions and mental retardation were common as compared to stroke, dementias and Parkinson’s disease [7].

The available information on the pattern and frequency of major neurological disorders in Pakistan is limited to hospital-based populations who may not be totally representative of the community. The incidence or prevalence of neurological diseases in Pakistan is not known [8]. However the Global Burden of Disease 2010 data suggests that more than half of disability due to neurological diseases is related to stroke followed by dementias, migraine, epilepsy and tetanus [9].

The estimated prevalence of people with dementia in Pakistan is around 200,000. The estimated volume of visits to neurology clinics in Pakistan is close to 200,000 visits per year. The demographics and diagnosis of these patients may vary from area to area. A data base of demographics and diagnosis of outpatients seen at neurology clinics at a tertiary care hospital not only identifies the frequency of different neurological diseases but also includes patient details such as gender and age groups. Findings from this database may open up avenues for further research, particularly in the development of community based studies and interventions/clinical trials to help improve the health of the population.

### Aims and objectives

The overall aim of the study was to generate knowledge about neurological disorders NDs in patients attending neurologic clinics in a low-income setting and to contribute the neglected public health field in Pakistan.

The main objective of the study was to estimate the frequency of the main NDs in outpatient neurological clinics at a tertiary care hospital in Karachi

## Main text

### Methods

#### Study design and setting

The records of all out-patients (18 years and above) seen in neurological clinics were collected at Aga Khan University (AKU) hospital from January 2014 to December 2015. A retrospective observational study was conducted of all presentations to neurology clinics over a period of 2 years. The study was approved by the Ethical Review Committee (ERC) of the Aga Khan University.

#### Data collection

The WHO protocol for the detection of major neurological disorders was used as the basis for the questionnaire. The questionnaire was tested and modified to suit the needs of this study. The questionnaire was designed to collect data on demographics including age, sex, place and type of residence (rural/urban), referral and patient’s evaluation status (new versus follow-up patient). A diagnosis as a referenced neurology case was determined by neurologist after conducting a physical examination of the patient. The main types of NDs along with sub-group were offered in the questionnaire to record the exact neurological disorder [10].

Uniform and standard criteria was used by neurologists for the diagnosis of cases. For each disorder, WHO classifications and definitions were used to assess the neurological disorder as follows. (1) Vascular diseases, (2) CNS infections, (3) demyelinating diseases, (4) movement disorders, (5) cerebellar ataxias and hereditary spastic paraplegias, (6) CNS neoplasms, (7) dementias, (8) epilepsies, (9) headache disorders, (10) nerve and root lesions, (11) myopathies/muscle disorders, (12) spinal disorders, (13) acquired metabolic and toxic disorders, (14) psychiatric disorders, (15) developmental disorders, and (16) neurometabolic/nutritional disorders of the nervous system.

The diagnoses were made clinically with laboratory and radiological confirmation. Radiological tests used included CT-scans (brain and spine), spine X-rays, and MRI. Electrophysiological tests included electroencephalography (EEG), electroneuromyography (ENMG), and electrocardiography (ECG).

#### Statistical analysis

Descriptive analysis for demographic variables was performed; results were reported as numbers with percentages for quantitative variables, and mean ± standard deviation or median with ranges for all qualitative variables. Comparison between different age groups and gender with neurological diseases were performed using Pearson Chi square test. *p* < 0.05 was considered as statistically significant; all *p* values were two sided. SPSS 19.0 (SPSS Inc., Chicago: IL, USA) was used for data entry and analysis.

### Results

A total of 16,371 out-patients with neurological diseases were seen during the study period. The mean age of the study participants was 46.2 ± 18.3 years with median of 46 years. Patients over 40 years made up 8389 (51.2%) of the participants. A total of 8508 (52%) were male and 7867 (48.1%) were female.

**Fig. 1:**
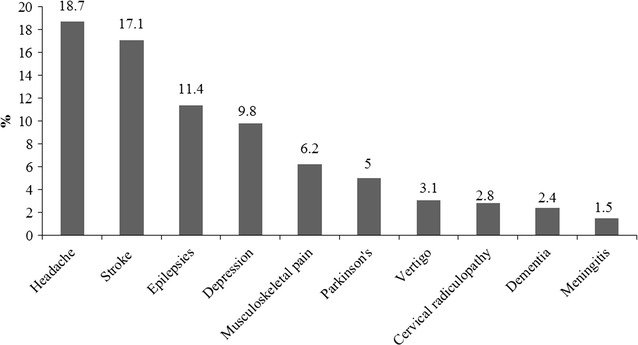
The ten leading diseases in the out-patients neurology clinics

The frequency of various neurological disorder groups is shown in Table 1. Headache disorders were present in 18.6% of patients followed by vascular diseases 17.4%, nerve and root lesions 14.1%, epilepsies 12.5%, psychiatric disorders 11.7% and neurometabolic/nutritional disorders 11.3%. Among the vascular group stroke was presented in 2792 (98.2%) of patients. The most common sub type was ischaemic stroke 2277 (81.6%) followed by haemorrhagic stroke 294 (10.5%). Migraine 1698 (55.5%) was the most common presentation amongst the headache disorder group. The most common presentation among the sleep disorders group was obstructive sleep apnoea 105/195 (53.8%) followed by restless leg syndrome 87/195 (44.6%). Among the CNS infections group, tuberculous meningitis 162 (65.3%), viral meningitis 44 (17.7%) and bacterial meningitis 32 (12.9%) were the most common presentations. The dementia group is mainly constituted of two types of diseases; Alzheimer’s 211 (45.8%) followed by dementia 207 (45%). Among movement disorders, Parkinson’s disease 821 (67.7%) was the most common presentation, followed by dystonia 141 (11.6%). Partial epilepsy with or without generalization 629 (44.9%) was the most common type of epilepsies followed by generalized epilepsy 373 (26.6%) and epilepsy 326 (23.3%). Musculoskeletal pain 1017 (58.6%) was the most common presentation of myopathies. Vertigo was seen in 510 (3.11%) patients followed by Bell’s palsy 161 (0.98%). The frequency of the top neurological diseases seen in out-patient clinics are presented in Fig. 1. The frequencies of neurological diseases in patients above the age of 45 years are presented in Additional file 1. The prevalence of stroke was similar in both genders. Parkinson’s disease was more prevalent in males 564 (70.8%) than females 257 (62.1%); (*p* = 0.002). Epilepsies were seen more in males 737 (71.3%) as compared to females 665 (65.1%); (*p* = 0.003). Migraines 1231 (60.7%) versus 467 (45.4%) and vertigo disease 260 (61.6%) versus 249 (49.7%) were more common in females as compared to males; Table 2. Of the Parkinson’s disease patients 50.9% were between the ages of 45 and 65 years. A comparison of different disease and age groups is shown in Additional file 2.

**Table 1:** Diagnosis profile of the study population n = 16,371

Diagnosis	n	%
*Vascular disease*	2842	17.4
Stroke	2792/2842	98.2
Types (n = 2729)		
Ischaemic stroke	2277	81.6
Transient stroke	170	6.1
Haemorrhagic stroke	294	10.5
Ischemia and hemorrhagic	19	0.7
Ischemia and transient	30	1.1
Transient and hemorrhage	2	0.1
*Cerebellar ataxias and hereditary spastic paraplegias*	311	1.9
Motor neuron diseases	60	19.2
*Sleep disorders*	195	
Restless leg syndrome	87	44.6
Obstructive sleep apnoea	105	53.8
*Headache disorders*	*3058*	18.6
Type of headache (n = 1370)		
Headache disorder	651	21.2
Tension type headache	574	18.7
Cluster headache	29	0.68
Chronic headache	28	2.04
Headache due to psychiatric disorder	26	1.89
Headache attributed to head and/or neck trauma	11	0.35
Migraine	1698/3058	55.5
*Acquire metabolic and toxic disorders*	13	0.07
*CNS infection*	314	0.01
*Meningitis* (n = 248)		
Bacterial meningitis	32	12.9
Tuberculous meningitis/tuberculoma	162	65.3
Fungal meningitis	6	2.4
Viral meningitis	44	17.7
Symptomatic meningitis	4	1.6
*CNS neoplasm*	151	0.92
Meningioma	27	17.8
Oligodendroglioma	23	15.2
Pituitary adenoma	20	13.2
Glioma	15	9.9
*Nerve and root lesion*	2311	14.1
Cervical radiculopathy	369	15.9
Carpal tunnel syndrome	153	6.6
Diabetic polyneuropathy	129	5.5
Guillain–Barre syndrome(AIDP)	94	4.0
*Psychiatric disorders*	1916	11.7
Depression	1486	77.5
Anxiety	188	9.8
Conversion disorders	86	4.4
*Demyelinating diseases*	181	1.10
Multiple sclerosis	121/181	6.6
*Dementias*	460	2.8
Alzheimer’s diseases	211	45.8
Dementia frontal/vascular	207	45
*Myopathies/muscle disorders*	*1734*	10.5
Musculoskeletal pain	1017	58.6
Myasthenia gravis	190	10.9
Fibromyalgia	193	11.1
Myopathies/muscle disorders	49	2.8
Neck strain	39	2.2
Polymyositis	33	1.9
*Development disorders*	*81*	0.49
Mental retardation	19	23.4
*Movement disorders*	*1211*	7.3
Parkinson’s diseases	821	67.7
Dystonias	141	11.6
Essential tremor	89	7.3
*Epilepsies*	*2055*	12.5
Epilepsy	326	23.3
Partial epilepsy with or without generalization	629	44.9
Generalised epilepsy	373	26.6
Juvenile myoconic epilepsy	55	3.9
*Seizures*	591	28.7
Febrile seizures	7	1.2
Seizures vs. Pseudoseizures	13	2.2
Fits	91	15.4
Complex partial seizures	3	0.5
Seizures	439	74.3
GTC (general tonic chronic seizures)	16	2.7
Pseudoseizures	12	2.7
Non-epileptic seizures	2	2.0
Post traumatic seizures	3	0.5
Systematic seizures	1	0.2
Neonate seizure	1	0.2
Focal seizures	2	0.3
Syncopy vs. seizures	1	0.2
*Spinal disorders*	124	0.75
Myelitis	62	50
Myelopathy	62	50
*Others*		
Bell palsy	161	0.98
Vertigo	510	3.11
Encephalopathy	33	0.20
*Neurometabolic/nutritional*	174	1.06
Vitamin D deficiency osteomalacic	72	41.3
B12 deficiency	80	45.9
Wilson disease	22	12.6

**Table 2:** Distribution of diagnosis among male and females

	Male, n (%)	Female, n (%)	*p* value
Stroke, n = 2792	1795 (98.6)	997 (97.6)	0.052
Meningitis, n = 248	127 (78.4)	121 (80.1)	0.78
Parkinson’s disease, n = 821	564 (70.8)	257 (62.1)	0.002
Epilepsy, n = 1402	737 (71.3)	665 (65.1)	0.003
Musculoskeletal pain, n = 1017	471 (59.1)	546 (58.3)	0.73
Migraine, n = 1698	467 (45.4)	1231 (60.7)	< 0.001
Vertigo, n = 510	249 (49.7)	260 (61.6)	< 0.001
Alzheimer’s, n = 211	106 (43.8)	105 (48.2)	0.35
Dementia, n = 207	104 (43)	103 (47.2)	0.39
Obstructive sleep apnoea, n = 105	60 (33.3)	45 (34.4)	0.90

### Discussion

The study was designed to investigate the frequency of NDs in Pakistan. NDs commonly cause public health challenges, creating burdens and disability.

In our study, the age group between 46 and 65 years represented 34% of all out-patient visits. A study from Dhaka, Bangladesh reported 59.7% of admissions in neurology were after the age of 40 years [11]. A study from Nigeria reported 40.8% were aged between 51 and 60 years [12]. The mean age of the study participants was 46 years which was similar to the mean age reported in other studies done in Pakistan [13], Bangladesh [11] and African countries [14–16]. Most patients were self-referrals and the leading diagnosis in our study were headache, stroke, epilepsies, depression, musculoskeletal pain, Parkinson’s, vertigo, cervical radiculopathy, dementia and meningitis. The leading cause of headache disorders is similar to the study by Alam et al. conducted in Peshawar, Pakistan [13], Anand et al. from India [17], and Callixte et al. from sub-Saharan Africa [16, 18].

The most common cause of out-patient visits was stroke (98.2%), which is similar to previous studies [11, 12, 14, 16, 19]. According to WHO estimates in 2001, 86% of deaths related to stroke worldwide occurred in developing countries [20].

A population-based study [21] from Pakistan showed a much higher prevalence of stroke 19,000 per 100,000 population (19·1%) in comparison with worldwide literature. A study from India estimates the prevalence of stroke ranges from 44 to 843 per 100,000 population [22]. Further studies are needed to determine the prevalence of stroke subtypes between Pakistani and Western populations [23]. Furthermore, meningitis was the second leading cause of out-patient clinic visit in our study. A prior study reported headache as the most common presentation, followed by cerebrovascular accidents and epilepsy [13]. A study from Bangladesh reported that stroke and seizures were the most seen diagnosis [11]. Other than meningitis, epilepsy was the most common neurological disorder and the highest prevalence is seen in people under 30 years of age [24]. Approximately 50 million people worldwide have epilepsy, making it one of the most common neurological disorders [25].

Neurodegenerative diseases are frequent in older populations. Meta-analysis of worldwide data showed the prevalence of Parkinson’s disease rising with age. An estimated 7 to 10 million people are living with Parkinson’s disease [26]. Prevalence differed by geographic location [27], however, dementia was seen both in young and older age groups [28]. The prevalence of dementia in South Asia is estimated to be 1.9% [29]. Alzheimer’s disease is responsible for 45.8% of admissions in our neurological setting.

Mental illnesses in Pakistan depicts a gloomy picture with a 6% prevalence of depression, 1.5% schizophrenia, 1–2% epilepsy and 1% of Alzheimer’s disease [30]. Due to a lack of research, it is difficult to get an accurate number of people suffering from these diseases.

Pakistan’s estimated population is over 194.9 million, making it one of the world’s most-populous countries. At least 10% of the population of Pakistan suffers from neurological diseases. More than 70% of medical colleges and almost 75% of government hospitals are without neurologists. Pakistan produces 10–20 neurologists annually out of which 50% leave the country. The present study lays a foundation of data regarding ND burden in this region to build a registry of data and address the gap in existing information about this neglected area of disease epidemiology.

### Limitation

We had some limitations in this study. First, this study is a hospital-based design and patients were seen only once by the neurologist so follow up data was unavailable. Second, this study was conducted in an urban centre so the data is not necessarily representative of the whole country. Further studies involving large cohorts are required to validate these findings in both urban and rural populations.

## Additional files

**Additional file 1.** The ten leading diseases in the out-patient neurology clinics. Leading diseases among patients above the age of 45 years.

**Additional file 2.** Age wise distribution of diagnosis. Description of data: age wise distribution of leading diagnosis.

**Additional file 3.** Demographic and clinical characteristics.

## Abbreviations

NDs: neurological disorders
DALYs: disability-adjusted life-years
ECG: electrocardiography
