# Supplementary figures and images for: Pattern of neurological diseases in adult outpatient neurology clinics in tertiary care hospital
Source: BMC Res Notes. 2017 Nov 2;10:545. doi: 10.1186/s13104-017-2873-5 (PMC5667470; doi:10.1186/s13104-017-2873-5)

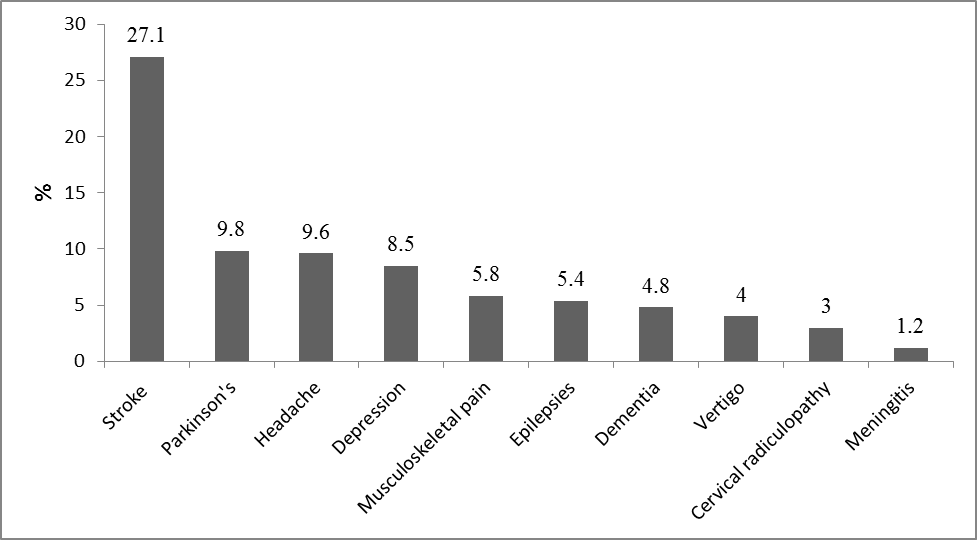


Figure 2: The leading disease in the out-patients above the age of 45 years in neurology clinics

Supplement: Supplementary file 1 — Additional file 1. The ten leading diseases in the out-patient neurology clinics. Leading diseases among patients above the age of 45 years. [file 13104_2017_2873_MOESM1_ESM.doc]
