# Supplementary material for: Pattern of neurological diseases in adult outpatient neurology clinics in tertiary care hospital
Source: BMC Res Notes. 2017 Nov 2;10:545. doi: 10.1186/s13104-017-2873-5 (PMC5667470; doi:10.1186/s13104-017-2873-5)
Supplement: Supplementary file 2 — Additional file 2. Age wise distribution of diagnosis. Description of data: age wise distribution of leading diagnosis. [file 13104_2017_2873_MOESM2_ESM.doc]

**Table 3**: Age wise distribution of diagnosis

|  | **<45 years** | **45-65** | **>65 years** | ***p* value** |
| --- | --- | --- | --- | --- |
| Headache (Migraine=1698) | 2252(73.6) | 687(22.5) | 119(3.9) | <0.001 |
| Stroke | 518(18.6) | 1353(48.5) | 921(33) | <0.001 |
| Epilepsies (seizures=591) | 1421(75.8) | 318(17) | 135(7.2) | <0.001 |
| Meningitis | 149(60.1) | 65(26.1) | 34(13.7) | 0.002 |
| Cervical radiculopathy | 211(46) | 203(44.2) | 45(9.8) | <0.001 |
| Depression (anxiety=188) | 888(55.4) | 551(34.4) | 164(10.2) | <0.001 |
| Musculoskeletal pain | 534(52.5) | 391(38.4) | 92(9.0) | <0.001 |
| Vertigo | 174(34.2) | 229(45) | 106(20.8) | <0.001 |
| Parkinson’s disease | 0 | 418(50.9) | 403(49.1) | <0.001 |
| Dementia (Alzheimer’s=211) | 0 | 120(29.9) | 281(70.1) | <0.001 |
| Obstructive sleep apnoea | 35(33.3) | 51(48.6) | 19(18.1) | 0.003 |
|  |  |  |  |  |
