# Supplementary material for: Pattern of neurological diseases in adult outpatient neurology clinics in tertiary care hospital
Source: BMC Res Notes. 2017 Nov 2;10:545. doi: 10.1186/s13104-017-2873-5 (PMC5667470; doi:10.1186/s13104-017-2873-5)
Supplement: Supplementary file 3 — Additional file 3. Demographic and clinical characteristics. [file 13104_2017_2873_MOESM3_ESM.doc]

Frequency Table
age_cat	
	Frequency	Percent	Valid Percent	Cumulative Percent	
Valid	16-30	3836	23.4	23.4	23.4	
	31-40	2800	17.1	17.1	40.5	
	41-50	2775	17.0	17.0	57.5	
	51-60	2865	17.5	17.5	75.0	
	61-70	2380	14.5	14.5	89.5	
	>70	1715	10.5	10.5	100.0	
	Total	16371	100.0	100.0		


Gender	
	Frequency	Percent	Valid Percent	Cumulative Percent	
Valid	Male	8508	52.0	52.0	52.0	
	Female	7863	48.0	48.0	100.0	
	Total	16371	100.0	100.0		


Refer	
	Frequency	Percent	Valid Percent	Cumulative Percent	
Valid	Self	6252	38.2	92.9	92.9	
	Physicain	352	2.2	5.2	98.1	
	Family and friend	127	.8	1.9	100.0	
	Total	6731	41.1	100.0		
Missing	System	9640	58.9			
Total	16371	100.0			


stroke_10 * Gender
Crosstab	
	Gender	Total	
	Male	Female		
stroke_10	.00	Count	6713	6866	13579	
		% within Gender	78.9%	87.3%	82.9%	
	1.00	Count	1795	997	2792	
		% within Gender	21.1%	12.7%	17.1%	
Total	Count	8508	7863	16371	
	% within Gender	100.0%	100.0%	100.0%	


Chi-Square Tests	
	Value	df	Asymp. Sig. (2-sided)	Exact Sig. (2-sided)	Exact Sig. (1-sided)	
Pearson Chi-Square	204.711a	1	.000			
Continuity Correctionb	204.116	1	.000			
Likelihood Ratio	207.598	1	.000			
Fisher's Exact Test				.000	.000	
Linear-by-Linear Association	204.699	1	.000			
N of Valid Cases	16371					
a. 0 cells (.0%) have expected count less than 5. The minimum expected count is 1341.00.	
b. Computed only for a 2x2 table	

meningitis_10 * Gender
Crosstab	
	Gender	Total	
	Male	Female		
meningitis_10	.00	Count	8381	7742	16123	
		% within Gender	98.5%	98.5%	98.5%	
	1.00	Count	127	121	248	
		% within Gender	1.5%	1.5%	1.5%	
Total	Count	8508	7863	16371	
	% within Gender	100.0%	100.0%	100.0%	


Chi-Square Tests	
	Value	df	Asymp. Sig. (2-sided)	Exact Sig. (2-sided)	Exact Sig. (1-sided)	
Pearson Chi-Square	.058a	1	.809			
Continuity Correctionb	.031	1	.859			
Likelihood Ratio	.058	1	.809			
Fisher's Exact Test				.848	.430	
Linear-by-Linear Association	.058	1	.809			
N of Valid Cases	16371					
a. 0 cells (.0%) have expected count less than 5. The minimum expected count is 119.11.	
b. Computed only for a 2x2 table	


Parkinson_10 * Gender
Crosstab	
	Gender	Total	
	Male	Female		
Parkinson_10	.00	Count	7944	7606	15550	
		% within Gender	93.4%	96.7%	95.0%	
	1.00	Count	564	257	821	
		% within Gender	6.6%	3.3%	5.0%	
Total	Count	8508	7863	16371	
	% within Gender	100.0%	100.0%	100.0%	


Chi-Square Tests	
	Value	df	Asymp. Sig. (2-sided)	Exact Sig. (2-sided)	Exact Sig. (1-sided)	
Pearson Chi-Square	96.883a	1	.000			
Continuity Correctionb	96.179	1	.000			
Likelihood Ratio	99.564	1	.000			
Fisher's Exact Test				.000	.000	
Linear-by-Linear Association	96.877	1	.000			
N of Valid Cases	16371					
a. 0 cells (.0%) have expected count less than 5. The minimum expected count is 394.33.	
b. Computed only for a 2x2 table	
Epilepse_10 * Gender
Crosstab	
	Gender	Total	
	Male	Female		
Epilepse_10	0	Count	7556	6941	14497	
		% within Gender	88.8%	88.3%	88.6%	
	1	Count	952	922	1874	
		% within Gender	11.2%	11.7%	11.4%	
Total	Count	8508	7863	16371	
	% within Gender	100.0%	100.0%	100.0%	


Chi-Square Tests	
	Value	df	Asymp. Sig. (2-sided)	Exact Sig. (2-sided)	Exact Sig. (1-sided)	
Pearson Chi-Square	1.160a	1	.282			
Continuity Correctionb	1.107	1	.293			
Likelihood Ratio	1.159	1	.282			
Fisher's Exact Test				.291	.146	
Linear-by-Linear Association	1.160	1	.282			
N of Valid Cases	16371					
a. 0 cells (.0%) have expected count less than 5. The minimum expected count is 900.08.	
b. Computed only for a 2x2 table	


muslepain_10 * Gender
Crosstab	
	Gender	Total	
	Male	Female		
muslepain_10	.00	Count	8037	7317	15354	
		% within Gender	94.5%	93.1%	93.8%	
	1.00	Count	471	546	1017	
		% within Gender	5.5%	6.9%	6.2%	
Total	Count	8508	7863	16371	
	% within Gender	100.0%	100.0%	100.0%	


Chi-Square Tests	
	Value	df	Asymp. Sig. (2-sided)	Exact Sig. (2-sided)	Exact Sig. (1-sided)	
Pearson Chi-Square	13.903a	1	.000			
Continuity Correctionb	13.663	1	.000			
Likelihood Ratio	13.893	1	.000			
Fisher's Exact Test				.000	.000	
Linear-by-Linear Association	13.903	1	.000			
N of Valid Cases	16371					
a. 0 cells (.0%) have expected count less than 5. The minimum expected count is 488.47.	
b. Computed only for a 2x2 table	


vertigo_10 * Gender
Crosstab	
	Gender	Total	
	Male	Female		
vertigo_10	.00	Count	8259	7603	15862	
		% within Gender	97.1%	96.7%	96.9%	
	1.00	Count	249	260	509	
		% within Gender	2.9%	3.3%	3.1%	
Total	Count	8508	7863	16371	
	% within Gender	100.0%	100.0%	100.0%	


Chi-Square Tests	
	Value	df	Asymp. Sig. (2-sided)	Exact Sig. (2-sided)	Exact Sig. (1-sided)	
Pearson Chi-Square	1.958a	1	.162			
Continuity Correctionb	1.834	1	.176			
Likelihood Ratio	1.957	1	.162			
Fisher's Exact Test				.163	.088	
Linear-by-Linear Association	1.958	1	.162			
N of Valid Cases	16371					
a. 0 cells (.0%) have expected count less than 5. The minimum expected count is 244.47.	
b. Computed only for a 2x2 table	
Migraine_10 * Gender
Crosstab	
	Gender	Total	
	Male	Female		
Migraine_10	.00	Count	8041	6632	14673	
		% within Gender	94.5%	84.3%	89.6%	
	1.00	Count	467	1231	1698	
		% within Gender	5.5%	15.7%	10.4%	
Total	Count	8508	7863	16371	
	% within Gender	100.0%	100.0%	100.0%	

Chi-Square Tests	
	Value	df	Asymp. Sig. (2-sided)	Exact Sig. (2-sided)	Exact Sig. (1-sided)	
Pearson Chi-Square	454.350a	1	.000			
Continuity Correctionb	453.257	1	.000			
Likelihood Ratio	466.501	1	.000			
Fisher's Exact Test				.000	.000	
Linear-by-Linear Association	454.322	1	.000			
N of Valid Cases	16371					
a. 0 cells (.0%) have expected count less than 5. The minimum expected count is 815.55.	
b. Computed only for a 2x2 table	


Alzemier_10 * Gender
Crosstab	
	Gender	Total	
	Male	Female		
Alzemier_10	.00	Count	8402	7758	16160	
		% within Gender	98.8%	98.7%	98.7%	
	1.00	Count	106	105	211	
		% within Gender	1.2%	1.3%	1.3%	
Total	Count	8508	7863	16371	
	% within Gender	100.0%	100.0%	100.0%	


Chi-Square Tests	
	Value	df	Asymp. Sig. (2-sided)	Exact Sig. (2-sided)	Exact Sig. (1-sided)	
Pearson Chi-Square	.257a	1	.612			
Continuity Correctionb	.192	1	.662			
Likelihood Ratio	.257	1	.612			
Fisher's Exact Test				.628	.331	
Linear-by-Linear Association	.257	1	.612			
N of Valid Cases	16371					
a. 0 cells (.0%) have expected count less than 5. The minimum expected count is 101.34.	
b. Computed only for a 2x2 table	


Dementia * Gender
Crosstab	
	Gender	Total	
	Male	Female		
Dementia	0	Count	138	115	253	
		% within Gender	57.0%	52.8%	55.0%	
	1	Count	104	103	207	
		% within Gender	43.0%	47.2%	45.0%	
Total	Count	242	218	460	
	% within Gender	100.0%	100.0%	100.0%	

Chi-Square Tests	
	Value	df	Asymp. Sig. (2-sided)	Exact Sig. (2-sided)	Exact Sig. (1-sided)	
Pearson Chi-Square	.846a	1	.358			
Continuity Correctionb	.682	1	.409			
Likelihood Ratio	.846	1	.358			
Fisher's Exact Test				.398	.204	
Linear-by-Linear Association	.844	1	.358			
N of Valid Cases	460					
a. 0 cells (.0%) have expected count less than 5. The minimum expected count is 98.10.	
b. Computed only for a 2x2 table	


sleep_apnoea * Gender


Crosstab	
	Gender	Total	
	Male	Female		
sleep_apnoea	0	Count	120	86	206	
		% within Gender	66.7%	65.6%	66.2%	
	1	Count	60	45	105	
		% within Gender	33.3%	34.4%	33.8%	
Total	Count	180	131	311	
	% within Gender	100.0%	100.0%	100.0%	


Chi-Square Tests	
	Value	df	Asymp. Sig. (2-sided)	Exact Sig. (2-sided)	Exact Sig. (1-sided)	
Pearson Chi-Square	.035a	1	.851			
Continuity Correctionb	.004	1	.947			
Likelihood Ratio	.035	1	.851			
Fisher's Exact Test				.904	.473	
Linear-by-Linear Association	.035	1	.852			
N of Valid Cases	311					
a. 0 cells (.0%) have expected count less than 5. The minimum expected count is 44.23.	
b. Computed only for a 2x2 table	


stroke_10 * age_2
Crosstab	
	age_2	Total	
	<45	45-65	>65y		
stroke_10	No	Count	7464	4218	1897	13579	
		% within age_2	93.5%	75.7%	67.3%	82.9%	
	Yes	Count	518	1353	921	2792	
		% within age_2	6.5%	24.3%	32.7%	17.1%	
Total	Count	7982	5571	2818	16371	
	% within age_2	100.0%	100.0%	100.0%	100.0%	


Chi-Square Tests	
	Value	df	Asymp. Sig. (2-sided)	
Pearson Chi-Square	1322.337a	2	.000	
Likelihood Ratio	1381.735	2	.000	
Linear-by-Linear Association	1270.120	1	.000	
N of Valid Cases	16371			
a. 0 cells (.0%) have expected count less than 5. The minimum expected count is 480.60.	


meningitis_10 * age_2
Crosstab	
	age_2	Total	
	<45	45-65	>65y		
meningitis_10	No	Count	7833	5506	2784	16123	
		% within age_2	98.1%	98.8%	98.8%	98.5%	
	Yes	Count	149	65	34	248	
		% within age_2	1.9%	1.2%	1.2%	1.5%	
Total	Count	7982	5571	2818	16371	
	% within age_2	100.0%	100.0%	100.0%	100.0%	


Chi-Square Tests	
	Value	df	Asymp. Sig. (2-sided)	
Pearson Chi-Square	12.944a	2	.002	
Likelihood Ratio	13.011	2	.001	
Linear-by-Linear Association	9.882	1	.002	
N of Valid Cases	16371			
a. 0 cells (.0%) have expected count less than 5. The minimum expected count is 42.69.	


Parkinson_10 * age_2
Crosstab	
	age_2	Total	
	<45	45-65	>65y		
Parkinson_10	No	Count	7982	5153	2415	15550	
		% within age_2	100.0%	92.5%	85.7%	95.0%	
	Yes	Count	0	418	403	821	
		% within age_2	.0%	7.5%	14.3%	5.0%	
Total	Count	7982	5571	2818	16371	
	% within age_2	100.0%	100.0%	100.0%	100.0%	


Chi-Square Tests	
	Value	df	Asymp. Sig. (2-sided)	
Pearson Chi-Square	1003.953a	2	.000	
Likelihood Ratio	1232.320	2	.000	
Linear-by-Linear Association	1003.020	1	.000	
N of Valid Cases	16371			
a. 0 cells (.0%) have expected count less than 5. The minimum expected count is 141.32.	


Epilepse_10 * age_2


Crosstab	
	age_2	Total	
	<45	45-65	>65y		
Epilepse_10	No	Count	6561	5253	2683	14497	
		% within age_2	82.2%	94.3%	95.2%	88.6%	
	Yes	Count	1421	318	135	1874	
		% within age_2	17.8%	5.7%	4.8%	11.4%	
Total	Count	7982	5571	2818	16371	
	% within age_2	100.0%	100.0%	100.0%	100.0%	

Chi-Square Tests	
	Value	df	Asymp. Sig. (2-sided)	
Pearson Chi-Square	622.247a	2	.000	
Likelihood Ratio	648.664	2	.000	
Linear-by-Linear Association	519.358	1	.000	
N of Valid Cases	16371			
a. 0 cells (.0%) have expected count less than 5. The minimum expected count is 322.58.	


muslepain_10 * age_2
Crosstab	
	age_2	Total	
	<45	45-65	>65y		
muslepain_10	No	Count	7448	5180	2726	15354	
		% within age_2	93.3%	93.0%	96.7%	93.8%	
	Yes	Count	534	391	92	1017	
		% within age_2	6.7%	7.0%	3.3%	6.2%	
Total	Count	7982	5571	2818	16371	
	% within age_2	100.0%	100.0%	100.0%	100.0%	


Chi-Square Tests	
	Value	df	Asymp. Sig. (2-sided)	
Pearson Chi-Square	51.364a	2	.000	
Likelihood Ratio	59.365	2	.000	
Linear-by-Linear Association	27.490	1	.000	
N of Valid Cases	16371			
a. 0 cells (.0%) have expected count less than 5. The minimum expected count is 175.06.	


vertigo_10 * age_2

Crosstab	
	age_2	Total	
	<45	45-65	>65y		
vertigo_10	No	Count	7808	5342	2712	15862	
		% within age_2	97.8%	95.9%	96.2%	96.9%	
	Yes	Count	174	229	106	509	
		% within age_2	2.2%	4.1%	3.8%	3.1%	
Total	Count	7982	5571	2818	16371	
	% within age_2	100.0%	100.0%	100.0%	100.0%	


Chi-Square Tests	
	Value	df	Asymp. Sig. (2-sided)	
Pearson Chi-Square	45.406a	2	.000	
Likelihood Ratio	46.107	2	.000	
Linear-by-Linear Association	31.006	1	.000	
N of Valid Cases	16371			
a. 0 cells (.0%) have expected count less than 5. The minimum expected count is 87.62.	


Migraine_10 * age_2


Crosstab	
	age_2	Total	
	<45	45-65	>65y		
Migraine_10	No	Count	6608	5269	2796	14673	
		% within age_2	82.8%	94.6%	99.2%	89.6%	
	Yes	Count	1374	302	22	1698	
		% within age_2	17.2%	5.4%	.8%	10.4%	
Total	Count	7982	5571	2818	16371	
	% within age_2	100.0%	100.0%	100.0%	100.0%	


Chi-Square Tests	
	Value	df	Asymp. Sig. (2-sided)	
Pearson Chi-Square	827.682a	2	.000	
Likelihood Ratio	972.113	2	.000	
Linear-by-Linear Association	781.703	1	.000	
N of Valid Cases	16371			
a. 0 cells (.0%) have expected count less than 5. The minimum expected count is 292.28.	


Alzemier_10 * age_2


Crosstab	
	age_2	Total	
	<45	45-65	>65y		
Alzemier_10	No	Count	7981	5516	2663	16160	
		% within age_2	100.0%	99.0%	94.5%	98.7%	
	Yes	Count	1	55	155	211	
		% within age_2	.0%	1.0%	5.5%	1.3%	
Total	Count	7982	5571	2818	16371	
	% within age_2	100.0%	100.0%	100.0%	100.0%	


Chi-Square Tests	
	Value	df	Asymp. Sig. (2-sided)	
Pearson Chi-Square	499.049a	2	.000	
Likelihood Ratio	417.734	2	.000	
Linear-by-Linear Association	416.889	1	.000	
N of Valid Cases	16371			
a. 0 cells (.0%) have expected count less than 5. The minimum expected count is 36.32.	


Dementia * age_2
Crosstab	
	age_2	Total	
	<45	45-65	>65y		
Dementia	0	Count	6	70	177	253	
		% within age_2	100.0%	51.1%	55.8%	55.0%	
	1	Count	0	67	140	207	
		% within age_2	.0%	48.9%	44.2%	45.0%	
Total	Count	6	137	317	460	
	% within age_2	100.0%	100.0%	100.0%	100.0%	


Chi-Square Tests	
	Value	df	Asymp. Sig. (2-sided)	
Pearson Chi-Square	5.843a	2	.054	
Likelihood Ratio	8.104	2	.017	
Linear-by-Linear Association	.000	1	.992	
N of Valid Cases	460			
a. 2 cells (33.3%) have expected count less than 5. The minimum expected count is 2.70.	


sleep_apnoea * age_2


Crosstab	
	age_2	Total	
	<45	45-65	>65y		
sleep_apnoea	0	Count	81	76	49	206	
		% within age_2	69.8%	59.8%	72.1%	66.2%	
	1	Count	35	51	19	105	
		% within age_2	30.2%	40.2%	27.9%	33.8%	
Total	Count	116	127	68	311	
	% within age_2	100.0%	100.0%	100.0%	100.0%	


Chi-Square Tests	
	Value	df	Asymp. Sig. (2-sided)	
Pearson Chi-Square	4.021a	2	.134	
Likelihood Ratio	4.005	2	.135	
Linear-by-Linear Association	.001	1	.974	
N of Valid Cases	311			
a. 0 cells (.0%) have expected count less than 5. The minimum expected count is 22.96.	
